# Effectiveness of antidepressants in bipolar depression

**DOI:** 10.1192/j.eurpsy.2021.520

**Published:** 2021-08-13

**Authors:** S. Potanin, M. Morozova

**Affiliations:** Laboratory Of Psychopharmacology, FSBSI Mental Health Research Center, Moscow, Russian Federation

**Keywords:** bipolar depression, effectiveness, antidepressant treatment

## Abstract

**Introduction:**

Prescribing antidepressants in the treatment of bipolar depression remains highly controversial due to the inconsistency between routine clinical practice and the results of controlled trials.

**Objectives:**

To assess the validity of antidepressants use in bipolar depression from the point of view of evidence-based medicine.

**Methods:**

Database search (Scopus and MEDLINE) followed by analysis of studies concerning the efficacy and safety of antidepressants in the bipolar depression treatment.

**Results:**

The search found 23 studies. There was a high degree of inconsistency in the results, apparently related to the methodology. Only two studies compared the effectiveness of antidepressants in monotherapy with placebo. No differences were found in the study with 740 participants but in the study with 70 participants with type 2 bipolar disorder antidepressants were found to be more effective than placebo. Nevertheless, both studies had significant methodological issues. In 6 studies comparing the effectiveness of the combination of antidepressants with mood stabilizers against the combination of mood stabilizers with placebo, only the effectiveness of fluoxetine in combination with olanzapine was confirmed, other antidepressants were ineffective. At the same time, studies where antidepressants were compared with each other in combination with mood stabilizers revealed a significant clinical response to therapy. Risk of the treatment emergency adverse events were relatively low for SSRI.

**Conclusions:**

Despite the contradictory literature data, the use of antidepressants in bipolar depression is justified from the point of view of evidence-based medicine for certain groups of patients with taking into account risk factors.

